# Progress on the Regulation of the Host Immune Response by Parasite-Derived Exosomes

**DOI:** 10.3390/pathogens13080623

**Published:** 2024-07-26

**Authors:** Xinyue Zhang, Chuanxin Yu, Lijun Song

**Affiliations:** National Health Commission Key Laboratory of Parasitic Disease Control and Prevention, Jiangsu Provincial Key Laboratory on Parasite and Vector Control Technology, Jiangsu Provincial Medical Key Laboratory, Jiangsu Institute of Parasitic Diseases, Wuxi 214064, China; zhangxinyue@jipd.com

**Keywords:** exosomes, parasites, hosts, immune responses

## Abstract

Exosomes are membrane-bound structures released by cells into the external environment that carry a significant amount of important cargo, such as proteins, DNA, RNA, and lipids. They play a crucial role in intercellular communication. Parasites have complex life cycles and can release exosomes at different stages. Exosomes released by parasitic pathogens or infected cells contain parasitic nucleic acids, antigenic molecules, virulence factors, drug-resistant proteins, proteases, lipids, etc. These components can regulate host gene expression across species or modulate signaling pathways, thereby dampening or activating host immune responses, causing pathological damage, and participating in disease progression. This review focuses on the means by which parasitic exosomes modulate host immune responses, elaborates on the pathogenic mechanisms of parasites, clarifies the interactions between parasites and hosts, and provides a theoretical basis and research directions for the prevention and treatment of parasitic diseases.

## 1. Introduction

Exosomes are vesicle-like structures found outside cells, typically consisting of a lipid bilayer membrane structure ranging from 40 to 160 nm in diameter. Multivesicular bodies (MVBs) inside cells release these vesicles into the extracellular environment upon fusion with the plasma membrane where they form exosomes [1]. These vesicles contain various substances, including proteins, DNA, mRNA, noncoding RNA, lipids, metabolites, etc. [2]. Exosomes deliver these contents to target recipient cells, thereby mediating intercellular communication upon internalization by recipient cells [3]. The exosomal contents regulate the functions of target cells through paracrine and autocrine mechanisms [4].

At present, the preparation method for exosomes is still mainly based on differential centrifugation, and the detection of protein and lipid markers remains the most commonly used characterization method [5]. However, there are certain differences in protein expression between parasites and mammals, and further extensive research is needed to determine the selection of exosomal markers in different parasites. Additionally, confirmation of successful exosome isolation requires a series of techniques such as electron microscopy observation, size analysis, and flow cytometry [6].

Parasites such as protozoa, helminths, and arthropods have been found to produce exosomes, and parasite-derived exosomes modulate the interactions between parasites and hosts to evade or activate host immune responses, contributing to disease progression. This review summarizes recent research on the means by which parasite-derived exosomes regulate host immune responses.

## 2. Formation and Function of Parasite-Derived Exosomes

Parasite-derived exosomes can be released by almost all cells. Cells undergo membrane budding to form multiple vesicles, which are released into the extracellular matrix by the fusion of the vesicular outer membrane with the cell membrane [1]. Once released externally, these vesicles bind and fuse with target cells via endocytosis [7], thereby opening or degrading the vesicular membrane within the target cell and subsequently releasing its contents to exert regulatory effects [8].

The life cycles of parasites are complex, and parasites can release exosomes at different life stages. These vesicles released by parasitic pathogens or infected cells carry parasitic nucleic acids, antigens, virulence factors, drug-resistant proteins, proteases, and lipids [9,10] and play important roles in modulating host immune responses. The lipid bilayer structure of exosomes prevents the degradation of RNA and DNA by nucleases, increasing the stability of the carried nucleic acids. Exosomes can enhance the efficiency of nucleic acid delivery to target cells and tissues, thereby facilitating the rapid entry of nucleic acids into cells to exert immunoregulatory effects [11,12]. These vesicles also contain antigens and proteins that promote antigen presentation and work together to modulate host immune responses [13]. The delivery of invasive virulence and drug-resistant proteins via exosomes can increase parasite toxicity, aiding in parasite survival within the host. Exosomal proteases regulate the expression of relevant proteins in the host’s innate immune system, allowing parasites to evade host immune responses [14,15]. Exosomal lipids activate immune cells and participate in disease progression by binding to specific receptors [16]. Exosomes serve as important carriers of these signaling molecules and play a crucial role in communication between hosts and parasites.

## 3. Regulation of the Host Immune Response by Parasite-Derived Exosomes

### 3.1. Protozoa

#### 3.1.1. *Plasmodium* Exosomes Regulate Host Immune Response

After infecting a human body, malarial parasites can cause severe parasitic disease. Malaria can result in symptoms such as intermittent fever, vomiting, fatigue, and headaches caused by damage to red blood cells. Due to the use of preventive measures and the widespread use of various antimalarial drugs, the incidence and mortality rates of malaria have been effectively reduced. However, the disease still has a high fatality rate [17,18]. Because of the presence of *Plasmodium* parasites within red blood cells, research on the effects of *Plasmodium* exosomes has focused mostly on exosomes produced by *Plasmodium* infection of red blood cells.

Studies have revealed statistically significant differences in the diameters of exosomes from *Plasmodium falciparum* (*P. falciparum*)-infected red blood cells and those from uninfected red blood cells [19]. Researchers speculate that exosomes secreted by parasitized red blood cells activate macrophages through the Toll-like receptor 4 (TLR-4)/myeloid differentiation factor 88 (MyD88)-dependent pathway, inducing systemic inflammation. Using indirect immunofluorescence assays to detect the binding of antibodies against the *Plasmodium berghei* (*P. berghei*) ANKA strain to exosomes, it has been confirmed that these exosomes contain a large amount of *Plasmodium*-derived proteins. Compared with intact parasitized red blood cells, these exosomes have greater proinflammatory effects [20]. Research has shown that these exosomes are derived from intracellular parasites rather than being shed from the surface of red blood cell membranes [21,22].

In extracellular experiments, the exosomes of *P. falciparum* can be internalized by monocytes and macrophages, delivering their biomolecular cargo into the cytoplasm and triggering innate immunity. Exosomes from *Plasmodium* containing malarial DNA can enter innate immune cells and activate the STING/tbk1/irf3 signaling pathway [23]. A stimulator of interferon genes (STING), a cytosolic adaptor protein activated by DNA-binding proteins, is closely involved in the production of interferon 1 (IFN-1) in innate immune cells [24]. IFN-1 promotes parasite-specific T cell secretion of the immunosuppressive factor interleukin 10 (IL-10) by inhibiting the activity of innate immune cells and interferon-gamma (IFN-γ) production in specific CD4^+^ T cells, thereby aiding in controlling pathological damage to the immune system caused by acute malaria [25,26]. Exosomes from *P. falciparum* activate microglia derived from human monocytes, increasing the gene expression levels of the inflammatory cytokine tumor necrosis factor alpha (TNF-α), reducing IL-10 expression, and enhancing neuroinflammation in cerebral malaria [27]. Exosomes secreted by *P. berghei*-infected red blood cells stimulate RAW264.7 mouse monocyte macrophages, resulting in significant increases in the mRNA expression levels of CD86, iNOS, TNF-α, IL-1β, and IL-6 and significant decreases in the gene expression levels of Bcl-6 and SOCS-1 [28]. In vivo experiments involving the injection of exosomes from *P. berghei*-infected red blood cells into mice led to increased proportions of CD68^+^-TREM-1^+^ macrophages in liver tissue and elevated levels of proinflammatory cytokines (such as iNOs, TNF-α, IL-1β, and IL-6), triggering an excessive proinflammatory response and promoting macrophage polarization toward the M1 phenotype, thereby exacerbating liver pathology induced by *Plasmodium*. Additionally, these exosomes increase the numbers of apoptotic cells, CD68^+^ cells, and CD86^+^ macrophages in the liver, leading to increased rates of peripheral parasitemia and experimental cerebral malaria occurrence [28].

By analyzing *Plasmodium*-infected red blood cell exosomes through proteomic analysis, 101 *Plasmodium* proteins were detected in a recent study. *P. falciparum* erythrocyte membrane protein 1 (PfEMP1), a transmembrane protein, is a key virulence factor of *Plasmodium* and is present only in exosomes derived from the ring form, the early stage of the parasite. Exosomal PfEMP1 influences the host immune response by regulating host defense, the stress response, and cytokine secretion [22]. The PfPTP2 protein plays a crucial role in the budding of *Plasmodium* vesicles and exosome release [29], but its role in regulating the host immune response remains unclear. Apart from those in mature red blood cells, proteomic analysis revealed the presence of *Plasmodium* antigens in the exosomes of malaria-infected reticulocytes in mice. These parasitic antigens include serine repeat antigens, merozoite surface proteins 1 and 9, proteases, heat shock proteins, and some hypothetical proteins. It is predicted that these proteins, involved in antigen processing and presentation, trigger the host immune response, leading to malarial pathogenesis [30].

#### 3.1.2. *Toxoplasma gondii* Exosomes Regulate Host Immune Response

Infection of an intermediate host by *Toxoplasma gondii* (*T. gondii*) can cause a group of zoonotic diseases known as toxoplasmosis [31]. Proteomic analysis of *T. gondii*-secreted exosomes revealed an abundance of classic exosomal proteins, including HSP70, CD63, and P30 [32]. Further studies showed that *T. gondii* exosomes have an average size of 50 nm and stimulate macrophage activation by elevating the host levels of IFN-γ, IL-12, and TNF-α, thus triggering both humoral and cellular immune responses and playing a protective role against *T. gondii* infection [33]. Exosomes can carry excreted/secreted antigens (ESAs) produced by tachyzoites, promoting splenocyte proliferation and activation to produce cytokines. Research has confirmed that *T. gondii* and infected hosts can express the same microRNAs (miRNAs), with *miR-155-5p*, *miR-125b-5p*, and *miR-423-3p* being the most abundant miRNAs in *T. gondii* tachyzoites and their exosomes. Among these, *miR-155-5p* is involved in the activation of immune cells in a murine model of *T. gondii* infection by regulating T cell response differentiation, leading to the secretion of IFN-γ, and the cytokine IL-10 [34]; *miR-423-3p* was found to respond to the TNF-α inflammatory factor [35]; *miR-21-5p*, which is exclusively expressed in exosomes, can downregulate TLR-4 expression and inhibit the expression of proinflammatory cytokines, while *miR-125b-5p* is associated with inflammatory diseases and T cell differentiation [36].

#### 3.1.3. *Leishmania* Exosomes Regulate Host Immune Response

*Leishmania* is transmitted to humans through sandflies. Based on its clinical features, leishmaniasis can be categorized into cutaneous, mucocutaneous, and visceral forms [37]. *Leishmania* exosomes induce macrophages to secrete IL-8, affecting macrophage signaling and function, recruiting neutrophils, promoting inflammatory responses, and worsening disease pathology [38]. In addition to affecting macrophages and neutrophils, *Leishmania* exosomes may influence monocyte and dendritic cell immune responses. By promoting the production of IL-10 and suppressing TNF-α activation, *Leishmania* exosomes inhibit monocyte immunoreactivity to IFN-γ. Moreover, these exosomal vesicles can inhibit the secretion of TNF-α and IL-10 by dendritic cells in a dose-dependent manner, although the exact mechanisms involved remain unclear [39]. *Leishmania* exosomes contain the virulence protein surface metallopeptidase GP63, which antagonizes macrophage lysosomal destruction of parasitic *Leishmania* cells by cleaving proteins such as protein tyrosine phosphatases (PTPs) and transcription factors (TFs) [40]. Another protein found in *Leishmania* exosomes, elongation factor 1α (EF-1α), negatively regulates IFN-γ signaling and inhibits the macrophage response by downregulating the production of TNF-α and NO, thereby creating a parasite-conducive environment [41]. The exosomal chaperone GroES (CPN10) protein can inhibit macrophage uptake by *Leishmania* parasites while also reducing the expression of immune-related proteins in macrophages [42]. Another exosomal protein, *Leishmania infantum* eukaryotic initiation factor (LieIF), induces protective immunity, enhancing immune function and suppressing *Leishmania* growth [43].

#### 3.1.4. *Trypanosoma* Exosomes Regulate Host Immune Response

Trypanosomiasis is a parasitic disease caused by *Trypanosoma*, in which exosomes mediate the release of most excretory/secretory proteins, including abundant proteases that play a role in pathogenesis. *Trypanosoma* exosomes contain several flagellar proteins contributing to virulence and serum resistance-associated (SRA) proteins necessary for human infection. These enable the transfer of *Trypanosome* lytic factors (TLFs) from resistant to nonresistant trypanosomes via exosomes, thereby aiding in the evasion of host lytic factors and escape from human innate immunity [14]. Furthermore, *Trypanosoma* exosomes induce high expression of IL-4 and IL-10 in macrophages and CD4^+^ T-cells, leading to a shift in the host immune response from T helper (Th) 1 to Th2 [44]. All these mechanisms contribute to the growth and development of *Trypanosoma* in the host. Proteomic analysis revealed that *Trypanosoma* exosomal proteins are closely associated with parasite survival and virulence and participate in host processes such as metabolism, signaling, and nucleic acid binding [45]. Exosomal RNA can induce changes in the host cellular gene expression profile. For instance, tsRNAs in exosomes induce the overexpression of proinflammatory cytokines such as chemokine (C-X-C motif) ligand 2 (CXCL2) and activating transcription factor 3 (ATF3) in host cells, thereby regulating cellular immune responses [46].

#### 3.1.5. *Trichomonas vaginalis* Exosomes Regulate Host Immune Response

*Trichomonas vaginalis* (*T. vaginalis*) infection can cause trichomoniasis, with clinical manifestations such as itching, odor, and pain during sexual intercourse or urination [47]. *T. vaginalis* exosomes contain conserved vesicular proteins and parasite-specific RNAs and proteins. Proteins involved in parasite adherence can enhance the adherence of *T. vaginalis* to vaginal and prostatic epithelial cells and regulate the immune response of host cells. Additionally, *T. vaginalis* exosomes may inhibit the secretion of IL-8 from peripheral cervical cells, transmit regulatory molecules to the host, and suppress the migration of neutrophils to the site of infection, playing a key role in establishing chronic infection [48]. Another study suggested that trichomonal exosomes can induce macrophages to produce more IL-10, slightly increase IL-6 and TNF-α production, and inhibit IL-13 and IL-17 expression, indicating that parasite-derived exosomes play a regulatory role in cytokine expression, thus suppressing the inflammatory response in infected mice [49].

### 3.2. Helminths

#### 3.2.1. *Schistosomes* Exosomes Regulate Host Immune Response

Schistosomiasis is a parasitic disease caused by infection with schistosome, which is primarily prevalent in tropical and subtropical areas and poses significant health risks to humans. Blood flukes can lay from 300 to 3000 eggs per day. Some of the parasitic eggs may remain in the host liver, triggering an immune response and, ultimately, leading to granulomatous inflammation and hepatic fibrosis [50].

A total of 403 proteins were identified in the exosomes of *Schistosoma japonicum* (*S. japonicum*) adult worms. Bioinformatics analysis predicted that 3.7% of these proteins may be involved in immune processes [51]. The excretory-secretory proteins of schistosome adult worms are rich in nucleic acid-binding proteins. RNA-binding proteins, including eukaryotic translation factors, can regulate host gene expression and even inhibit cell apoptosis, suggesting a potential regulatory role of *S. japonicum* excretory-secretory proteins in parasite–host interactions and evasion of host immune responses [52]. Exosomes also contain numerous schistosome surface proteins, mostly immune-related factors, which may participate in inhibiting both innate and adaptive host immunity, thereby contributing to immune evasion and immune-dependent parasite growth [53]. These exosomes contain peptidases, signaling proteins, cell adhesive proteins (e.g., integrins), glutathione-S-transferase (GST), tetraspanins (TSPs), and calpain, the latter three of which have been demonstrated to be candidate vaccine molecules against infection [54,55,56]. TSPs are crucial molecules in cell–cell interactions and in the internalization of excretory-secretory vesicles, facilitating antigen presentation and the activation of naïve CD4^+^ T cells. TSP-MHC interactions and the recruitment of MHC-peptide complexes to the TSP microdomain promote the formation of MHC-II tetramers and enhance antigen presentation [13]. *Schistosoma mansoni* (*S. mansoni*) exosomes carry various proteases, including metalloproteases, cysteine proteases, and serine proteases, which play roles in signaling pathways mediated by excretory-secretory vesicles, anticoagulation, or elimination of bound IgG and complement. These proteases contribute to invasion, migration, nutrient acquisition, and immune modulation [10]. However, whether these exosomes truly participate in the immune evasion process of schistosomes in the human body remains to be further studied.

The miRNAs carried by schistosome adult and egg-derived exosomes can be transferred to mammalian target cells where they can exert cross-species regulatory effects. A total of 143 miRNAs were identified in *S. mansoni* adult exosomes. Of these miRNAs, bioinformatics analysis revealed that *sma-miR-125b* has more than 600 potential human gene targets, while *sma-bantam* has 39 potential human gene targets [57]. *Sma-miR-10* has been confirmed to target *MAP3K7*, thereby downregulating nuclear factor *kappaB* (NF-κB) activity, which in turn activates the transcription factor gata3 and the AP-1 component junb, subsequently inducing IL-4 expression and playing a crucial role in Th2 signal transduction. This may be a significant factor leading to a decrease in the Th2 response and the chronicity of schistosome infection in the host after 8 weeks [58]. Various miRNAs have been identified in the *S. japonicum* adult exosomes [51]. Among the miRNAs, *Sja-miR-2162* targets the transforming growth factor beta receptor III (TGF-βR3) gene in hepatic stellate cells (HSCs), upregulating the expression of α-smooth muscle actin (α-SMA) and collagen 1 and promoting liver fibrosis development [59]. *Sja-let-7* targets the collagen 1α2 chain (Col1α2) gene in HSCs, downregulating the activity of the TGF-β/Smad signaling pathway both intracellularly and extracellularly, thus reducing HSC activation and alleviating liver fibrosis [60]. The amount of research performed on miRNAs carried by the egg-derived exosomes of *S. japonicum* has been gradually increasing in recent years. Yiluo Wang and colleagues cultured *S. japonicum* eggs in vitro and discovered a new miRNA, *miRNA-33*, which upregulates the mRNA and protein expression levels of α-SMA, collagen 1, and TGF-β receptor I (TGF-βRI) in HSCs, promoting liver fibrosis [61]. Exosomal Sja-miR-71a from *S. japonicum* eggs targets Semaphorin 4D (Sema4D), thereby inhibiting the TGF-β1/SMAD and IL-13/STAT6 pathways and regulating the Th1/Th2/Th17 and Treg balances to inhibit liver fibrosis [62]. *Sja-miR-71a* can also inhibit the formation of macrophage and neutrophil extracellular traps (METs and NETs) through the Sema4D/PPAR-γ/IL-10 axis, evade immune responses, and resist attacks from macrophages and neutrophils, suggesting that schistosomes evade the host immune response by releasing exosomes. miRNAs such as *sja-miR-71a*, *sja-miR-2162*, and *sja-let-7*, among others, target key signaling molecules in host cells to regulate the host immune response. The specific mechanism by which miRNAs in schistosome adult and egg-derived exosomes regulate the host immune response is detailed in Table 1.

In addition to proteins and miRNAs, lipids derived from the exosomes of *S. mansoni* adult worms, such as lysophosphatidylcholine and prostaglandin D2, can activate eosinophils through Toll-like receptor 2 (TLR2) and prostaglandin D2 receptor 1 (PDG1), promoting the release of TGF-β to enhance liver fibrosis and tissue repair [62]. Studies have shown that prostaglandins are present in exosomes and can be transferred between two cell types. Therefore, the lipid-rich exosomes of *S. mansoni* act as activators of eosinophilic innate immune pattern recognition receptors (PRRs) on the cell membrane, promoting eosinophil activation, participating in parasite–host communication, and facilitating the formation of parasitic egg granulomas and liver fibrosis [65].

#### 3.2.2. *Echinococcus granulosus* Exosomes Regulate Host Immune Response

*Echinococcus granulosus* (*E. granulosus*), a causative agent of the zoonotic disease cystic echinococcosis, primarily infects intermediate hosts such as sheep; humans act as accidental intermediate hosts by ingesting parasitic eggs. Cystic echinococcosis is most prevalent in livestock-farming regions in China, such as Xinjiang, Tibet, and Qinghai, and significantly impacts resident lives and livestock development [66]. It has been observed that both the protoscoleces (PSCs) and hydatid fluid (HF) of *E. granulosus* possess exosomes that affect the levels of two key components, interferon regulatory factor 5 (IRF5) and CD14, in the lipopolysaccharide (LPS)/Toll-like receptor 4 (TLR4) pathway, and upregulate the expression of proinflammatory cytokines while downregulating the expression of anti-inflammatory factors [67]. Exosomes from PSCs induce the maturation and differentiation of bone marrow-derived dendritic cells (BMDCs) in mice, leading to increased expression of proinflammatory cytokines such as IL-12 and TNF-α. Treatment of the parasite with the drug praziquantel stimulates BMDCs through their exosomes, upregulating the gene expression of IL-6, IL-10, and TGF-α within the cells. Conversely, treatment with albendazole sulfoxide promotes the parasitic secretion of exosomes, enhancing the expression of the inflammatory factor IL-12; this demonstrates that the exosomes produced during the treatment of cystic echinococcosis can modulate host immune responses, thereby reinforcing the activation of dendritic cells with a proinflammatory phenotype to control the growth of *E. granulosus* [68].

Analyses of 1175 proteins derived from HF- and PCS-derived exosomes revealed 1026 unique proteins in HF exosomes, 38 unique proteins in PCS exosomes, and 111 proteins common to both [69]. The antigenic protein components of PCS-derived exosomes may be recognized by important PRRs in the innate immune system, such as C-type lectins [70]. The leucine-rich repeat (LRR) protein LRRC33 interacts with Toll-like receptors (TLRs), exerting inhibitory effects on NF-κB expression, AP-1 activation, and cytokine production [71]. Tapeworm-secreted T cell immunomodulatory protein (TIP) may play a role in regulating parasitic development and early host Th1 responses, promoting the release of IFN-γ from CD4^+^ T cells in mice, and exerting antiparasitic effects [72], and enzymes such as leukotriene A4 (LTA4) hydrolase and prostaglandin E2 9-ketoreductase have proinflammatory effects [68]. The enzyme-mediated synthesis of inflammatory factors is a key factor in the Th2-type immune response. A series of parasite-specific molecules have been identified in HF-derived exosomal extracts. Antigen B may inhibit host immune responses by downregulating innate immune reactions mediated by neutrophils and dendritic cells as well as T cell-dependent mechanisms, promoting parasite growth and long-term infection [73]. Additionally, researchers have found that exosomes from the parasite can be internalized by dendritic cells, inducing their maturation. This process leads to upregulation of CD86 expression and downregulation of MHCII molecule expression. Furthermore, bioinformatics analysis revealed that exosomes from *E. granulosus* in the *protoscolices* and *metacestode* stages contain B cell receptor-associated protein 29 (Bp29), a basigin protein that inhibits T cell proliferation, and membrane-associated proteins that restrict neutrophil recruitment and promote inflammatory mediator production. The basigin protein, a member of the immunoglobulin superfamily functioning as a major receptor, mediates chemotaxis of eosinophils, regulates lymphocyte reactivity by inhibiting T cell proliferation, and plays a role in inducing typical Th2 responses and modulating dendritic cell maturation to help parasites evade host immune surveillance [74].

Various parasite-derived exosomal miRNAs have been detected in the blood of echinococcosis patients. For instance, *egr-miR-71* from PCS-derived exosomes can be internalized by sheep peripheral blood mononuclear cells (PBMCs), exerting an inhibitory effect on sheep PBMC immune responses. Toll/interleukin-1 receptor domain-containing adapter protein (TIRAP), as the first adaptor protein interacting with TLR4 on the membrane, recruits MyD88 to form a complex, promoting the production of inflammatory cytokines. *Egr-miR-71* inhibits the production of proinflammatory cytokines IL-1α, IL-1β, and TNF-α by downregulating TIRAP levels [66]. Bioinformatic analysis predicted that the top 20 most abundant miRNAs in exosomes from PSC cells of *E. granulosus* may be involved in parasite–host interactions and the regulation of host immune responses. *Egr-miR-125-5p* and *egr-miR-10a-5p* share similar sequences with host miRNAs, suggesting that they may interact with target genes through conserved sequences [75]. *Egr-miR-277a-3p*, one of the most abundant miRNAs in PSC-derived exosomes, has been found to directly target NF-κB1 and may induce the expression of the proinflammatory cytokines IL-6 and TNF-α by increasing the p65/p50 ratio of the nuclear transcription factor NF-κB in BMDCs, favoring the mediation of Th1 immune response, leading to inflammatory damage in the body [76].

#### 3.2.3. *Clonorchis sinensis* Exosomes Regulate Host Immune Response

Adult *Clonorchis sinensis* (*C. sinensis*) resides in the biliary ducts of the host where it can cause liver damage [77]. Exosomes from *C. sinensis* adult worms impair the migratory abilities of monocytes. Further proteomic analysis revealed that the exosomes contained peroxiredoxin, cathepsin B, cathepsin L1, and worm defense molecules and exhibited potent immunomodulatory effects, such as the ability to induce M2 macrophage activation, downregulate host immune responses, and suppress inflammatory reactions [78]. The exosomes of *C. sinensis* can regulate the immune surveillance function of host intestinal epithelial cells to maintain the Th2 immune microenvironment necessary for parasite survival and reproduction. They are also rich in miRNAs, potentially playing a crucial role in parasite immune evasion [79]. Apart from those of adult worms, the exosomes of *C. sinensis* eggs contain 23 different proteins, while those of larvae contain 29 different proteins, including catalytic and structural proteins, as well as proteins involved in cellular transport and vesicle formation pathways; these contribute to resistance against host innate immune cells for pathogen clearance. These proteins play a role in the parasite’s nutrition acquisition and immune evasion, aiding in resisting the clearance of pathogens by the host’s innate immune cells [80].

#### 3.2.4. *Trichinella spiralis* Exosomes Regulate Host Immune Response

Humans become infected with *Trichinella spiralis* by consuming undercooked meat. *Trichinella* adult worms parasitize the intestinal mucosa, causing *diarrhea*, while the larvae invade skeletal muscles, leading to muscle pain [81]. Studies have shown that the exosomes of *larval Trichinella* parasites residing in muscles carry immune-modulating proteins, inhibiting the activation of human renal fibroblasts, downregulating the expression levels of α-SMA, collagen I, collagen IV, and collagen VI, increasing the expression of monocyte IL-10 and IL-6, possibly inducing M2b macrophage polarization through high expression of *miR-1-3p* and *let-7-5p*, enhancing macrophage secretion levels of anti-inflammatory factors Arginase-1 (Arg-1) and TGF-β, exerting a potential anti-inflammatory effect [82]. However, other research has demonstrated that the exosomes of larval *Trichinella* induce the upregulation of IL-1 expression in intestinal epithelial cells and the downregulation of IL-10, TGF-β, TLR-5, MUC-1, and MUC-2 expression, promoting cellular inflammation. This process leads to a decrease in the expression levels of zonula occludens-1 (ZO-1), claudin-3 (CLDN-3), and occludin (OCLN), affecting the tight junctions of intestinal epithelial cells [83]. *miR-153* in larval exosomes negatively regulates Bcl2 expression in intestinal epithelial cells or regulates the ERK/p38-MAPK and p53 signaling pathways to promote apoptosis. Cell apoptosis is an important protective mechanism in organisms and plays a crucial role in maintaining host intestinal barrier function. By manipulating host cell apoptosis, *Trichinella spiralis* can evade detection and clearance by the host immune system, promoting its parasitism within the host [84].

### 3.3. Arthropods

The study of arthropod exosomes has attracted increased attention in recent years. In 2018, researchers reported that *tick* and mosquito exosomes can promote the transmission of pathogens between arthropod and vertebrate cells [85,86].

#### 3.3.1. Mosquito Exosomes Regulate Host Immune Response

Zika virus and Dengue virus are two of the most common flaviviruses. Both are transmitted by mosquitoes and cause Zika fever and Dengue fever in humans, respectively. Exosomes from Dengue virus-infected mosquito cells are larger than those from uninfected cells and contain virus-like particles capable of infecting immature C6/36 cells, indicating a role in virus transmission [87]. Researchers have also identified the full-length genome and viral proteins of Dengue virus serotype 2 (DENV2) within exosomes released from mosquito cells, demonstrating the highly infectious nature of exosome-loaded DENV2 RNA and proteins. Furthermore, exosomes derived from mosquito cells infected with DENV2 have been shown to infect Aedes aegypti C6/36 cells, mouse monocyte-derived dendritic cells (Mo-DCs), human immortalized keratinocytes (HaCaT), and human umbilical vein endothelial cells (HUVECs), with detectable viral loads in all four types of secreted exosomes. This finding suggests that exosomes carrying DENV2 can transmit viral RNA and proteins from mosquito cells to human cells. It is speculated that DENV2/DENV3 RNA and proteins within exosomes may evade viral checkpoints through host-neutralizing or non-neutralizing antibodies, as non-neutralizing antiviral proteins (i.e., antibodies) facilitate virus entry into host cells by binding to cell membrane Fc receptors [88].

Bioinformatics analysis revealed that mosquito saliva-derived miRNAs may participate in host immune responses and inflammatory reactions by targeting host cell mRNAs. For instance, among the 20 most abundant miRNAs in *Anopheles coluzzii* saliva, 11 are identical or nearly identical to human miRNAs. Specifically, *miR-7-5p* downregulates the activity of the NF-κB pathway by targeting the NF-κB subunit RelA84 and indirectly inhibits NF-κB activity by downregulating the expression of RNF183, a ubiquitin ligase that promotes degradation of the NF-κB inhibitor IκBo5. Additionally, *miR-1-3p* inhibits the expression of the inflammatory chemokine chemokine ligand 2 (CCL2), reducing the recruitment of monocytes, memory T cells, and natural killer cells to sites of tissue damage or infection-induced inflammation [89]. Research has also shown that *Aedes aegypti* miRNAs (aae-miR-1-3p, aae-miR-14-3p, and aae-miR-1891-2-5p) target two common genes, nuclear factor of activated T cells 2 (NFATC2) and phospholipase C gamma 2 (PLCG2), which are involved in immune system dysregulation [90].

#### 3.3.2. Tick Exosomes Regulate Host Immune Response

Ticks can be classified into hard ticks and soft ticks based on their morphological features. They carry a wide variety of pathogens, mainly viruses (such as Nyamanini virus and tick-borne encephalitis virus), bacteria (such as *Rickettsia* and spirochetes), protozoa, and helminths. In China, longhorn ticks carry the largest number of tick-borne pathogens, followed by hard ticks, meadow ticks, and tiny spinose ear ticks [91]. Tick saliva is an important vector for disease transmission by ticks, and salivary gland-derived exosomes can modulate host immune responses. Research by Adela S. Oliva Chávez and colleagues revealed that injecting salivary gland-derived exosomes from soft ticks reduced spleen enlargement in mice with bacterial infection and decreased the levels of IFN-γ and TNF-α in mouse blood, while salivary gland-derived exosomes from deer ticks promoted *Rickettsia* infection in mammalian hosts [92]. Furthermore, tick salivary gland-derived exosomes regulate the aggregation of host immune cells and the secretion of cytokines. γδ T cells not only act as a bridge between innate and adaptive immunity but also play a crucial role in host resistance against tick-borne pathogens and are associated with wound healing [93]. In the absence of tick exosomes, the number of γδ T cells at the site of tick bites significantly increases [92]. Studies have also shown that human epidermal keratinocytes treated with tick salivary gland-derived exosomes increase IL-8 secretion while decreasing CXCL12 levels, potentially enhancing inflammation and necrosis and disrupting the barrier protective function of the skin; this suggests that tick saliva-derived exosomes delay wound healing at tick bite sites to facilitate tick feeding on host’s blood [94].

Research on the function of miRNAs in tick salivary gland-derived exosomes is relatively limited. The 3′ end of miRNA is often uridylated or adenylated. Studies have shown that the proportion of uridylated miRNAs in tick salivary gland exosomes is similar to that in saliva, leading researchers to speculate that miRNAs in tick saliva likely originate from exosomes [95,96]. Tick saliva plays a crucial role in transmitting tick-borne pathogens. Host target gene prediction and functional analysis revealed that the miRNAs in tick saliva primarily participate in biological processes such as signal transduction, mRNA transcription and gene expression regulation, synaptic regulation, immune response, angiogenesis, and vascular development. The proteins encoded by genes targeted by these miRNAs play critical roles in immune homeostasis, innate and adaptive immune signal transduction, macrophage function programming, T cell activation, etc. [97].

## 4. Summary and Prospects

Parasitic diseases have long been a significant challenge in the field of global public health. Understanding the interactions between parasites and hosts is essential for the prevention and control of parasitic diseases. Exosomes, as carriers of signals between parasites and hosts, regulate host immune responses, leading to host pathological damage that promotes disease progression or evasion of host immune responses, promoting parasite survival. The abundant specific proteins, RNAs, and lipids in parasite-derived exosomes provide important clues for studying the interactions between exosomes and host immune systems.

Exosomes of protozoa and helminth both contain virulence factors or immune-regulatory molecules of the parasite, such as *Leishmania* parasite GP63, which can inhibit T cell activation or induce apoptosis of immune effector cells, thus aiding evasion of host immune surveillance [1]. miRNA carried by *T. gondii* exosomes is associated with inflammatory diseases and T cell differentiation [49]. Schistosome exosomes carry various proteases, which contribute to invasion, migration, nutrient acquisition, and immune modulation [10]. However, studies have found that the specific mechanisms by which parasite exosomes regulate host immunity differ significantly. Protozoa mostly parasitize intracellularly, and exosomes released by infected cells can stimulate T cell activation by presenting antigens to antigen-presenting cells, which generally helps the host combat parasite infections. On the other hand, helminth parasitizes extracellularly, and unlike protozoa that induce acute inflammation and pathological reactions, exosomes derived from helminth typically promote chronic inflammation by inducing regulatory and anti-inflammatory immune responses, thereby sustaining long-term infections and achieving coexistence with the host [98,99].

Moreover, vaccination is the most important and cost-effective measure for controlling infectious diseases. Several studies confirm the feasibility of using exosomes derived from parasites as vaccines. Immunizing BALB/c mice with exosomes from *P. vivax* resulted in reduced disease severity and increased survival upon reinfection, which is associated with the ability of exosomes to induce antibody immune responses [30]. The 23 newly identified parasitic proteins from exosomes obtained after *P. vivax* infection in vivo can participate in crucial metabolic processes, regulate internal balance, induce T cell immune responses, and hold potential as candidate vaccines [100]. Exosomes adsorbed on alum adjuvants compared with *T. gondii* ESAs can elicit higher humoral and cellular immune responses and reduce toxoplasma cysts, suggesting the potential of using exosomes secreted by *Toxoplasma*-infected cells combined with alum adjuvants as candidate vaccines [101]. In addition, potential vaccine molecules have been isolated from the exosomes of schistosome [102]. Specific proteins and RNAs from different developmental stages of *S. japonicum* cercarial excretory-secretory products, which were used as vaccine candidates, would be beneficial for early prevention and treatment of schistosomiasis [103]. The studies above suggest that exosomes from parasites have the potential as candidate vaccines for preventing and controlling the spread and pathogenesis of parasitic diseases.

This review summarizes research on the regulatory effects of parasite-derived exosomes on host immune responses and contributes to a deeper understanding of the pathogenic mechanisms of parasites, elucidates the interactions between parasites and hosts, and provides a new theoretical basis and research directions for the prevention and treatment of parasitic diseases. However, the mechanisms of extracellular vesicle generation by parasites, their entry into host target cells, and their biological functions need further clarification. Furthermore, while the role of miRNAs in interspecies regulation within exosomes is currently a popular area of research, the abundant proteins and lipids in exosomes and their roles and mechanisms in regulating host immune responses and interactions between parasites and hosts require further in-depth study.

## Figures and Tables

**Table 1:** Mechanisms by which schistosome exosomal miRNAs regulate host immune responses.

miRNA	Source	Target Gene	Function
*sma-miRNA-10*	*S. mansoni* adult worms	MAP3K7	Downregulating MAP3K7 expression and subsequently lowering NF-κB activity [58]
*miRNA-33*	*S. japonicum* eggs	TGF-β receptor I (TGF-βRI)	Upregulating the expression of TGF-β RI, promoting the activity of the TGF-β/Smad signaling pathway [61]
*sja-miR-71a*	*S. japonicum* eggs	Sema4D	Downregulating Sema4D expression, inhibiting the TGF-β/Smad and IL-13/STAT6 signaling pathways [63]
			Inhibiting the Sema4D/PPAR-γ/IL-10 axis, downregulating the formation of METs and NETs [64]
*sja-miR-2162*	*S. japonicum* eggs	TGF-β receptor III (TGF-βR3)	Downregulating TGF-βR3 expression, TGF-β signal transduction [59]
*sja-let-7*	*S. japonicum* adult worms	Col1α2	Downregulating the activity of the TGF-β/Smad signaling pathway, thus reducing HSC activation and alleviating liver fibrosis [60]

## Data Availability

The original contributions presented in the study are included in the article, further inquiries can be directed to the corresponding author/s.
